# Evaluation of antispasmodic activity of different *Shodhit guggul* using different *shodhan* process

**DOI:** 10.4103/0250-474X.43005

**Published:** 2008

**Authors:** Rachana Kamble, Sadhana Sathaye, D. P. Shah

**Affiliations:** University Institute of Chemical Technology, N. Parekh Marg, Matunga, Mumbai-400 019, India

**Keywords:** Antispasmodic, *guggul*, isolated ileum, charcoal transit

## Abstract

According to ayurvedic texts *shodhan vidhi* is an important process which enhances the biological activity of a compound and reduces the toxicity at the same time. Before incorporating into formulations, guggul is processed using *Shodhan vidhi* involving different *shodhan dravyas* like gulvel, *gomutra, triphala, dashmul*. We have evaluated the antispasmodic activity of guggul on ileum of guinea pig and Wistar rats. The animals were sacrificed and ileum tissue of guinea pig and rat was isolated and tested for antispasmodic activity using different spasmogens like acetylcholine, histamine and barium chloride. It was observed that the different *shodhit guggul (shudha guggul)* i.e. processed using different *shodhan vidhi*, showed good antispasmodic activity as compared to *Ashudha guggul*. When acetylcholine was used as spasmogen, *gulvel* and *triphala shodhit guggul* showed good antispasmodic activity than other shodhit guggul. Thus *shodhan vidhi* enhances the therapeutic properties of *guggul*.

*Guggul, Commiphora mukul* (Indian bdellium Tree) is a small tree or shrub with spinescent branches. It is a gum resin obtained from incision of the bark. Ayurvedic literature is full of praises for *guggul* and its divine action^1^. The explanation of word *guggul* is *Gunjo vyadhegurdti rakshati* which means to give relief against different diseases. Indians know g*uggul* from ancient age as it is used in the treatment of many diseases^2^. G*uggul* is a resin and hence before incorporating it into different formulations it is required to be purified and detoxified. This process is called as *Shodhan vidhi*. *Shodhan vidhi* is important to remove unwanted and harmful effects of the resin and to increase useful therapeutic effects. *Guggul* is purified by two processes, *Samanya shudhi* (general detoxification) and *Vishesh Shudhi* (special detoxification). *Vishesh shodhit guggul* is purified using special substances (*vishesh dravyas*) like *gulvel, dashmul, triphala* and *gomutra.* The present study has been carried out using *guggul* processed using different *Shodhan vidhi*, to evaluate its antispasmodic activity on guinea pig and rat ileum.

*Guggul* samples detoxified using different *Shodhan vidhi* involving *Shodhan dravyas* like *gulvel, gomutra, triphala kwath, dashmul kwath* and distilled water. *Guggul* was manufactured and supplied by Shri Dhootpapeshwar Ltd., Panvel, India. In order to compare the activity of various processed *guggul* with a marketed preparation, Entostal was used as a standard drug, which is a polyherbal, ayurvedic preparation used as antispasmodic marketed by Om Pharmaceuticals Ltd, Bangalore. *Shodhan vidhi* was carried out according to ayurvedic texts. First *Samanya shodhan* was done using distilled water and then *Vishesh shodhan* was done using different *Shodhan dravyas*^3^.

Study was performed using healthy Wistar rats and guinea pigs of average weight of either sex. Approval for the use of animals was obtained from the Institutional Animals Ethics Committee constituted for the purpose. (Protocol No: UICT/PH/IAEC/0405/09).

Healthy Wistar Rats weighing 150-200 g or guinea pigs (weighing 300-500 g) were procured for the study (Haffkines Institute, Mumbai). Animals of either sex were fasted 24 h before the study. Then the animals were sacrificed to isolate the ileum pieces. In case of rats, ether was used as anaesthetic agent, until death and guinea pigs were sacrificed by stunning or exsanguination as per CPCSEA recommended guidelines. Two methods were used to evaluate the Antispasmodic activity.

The *in vitro* method was performed using rat or guinea pig ileum using matching and interpolation method. Guinea pig ileum was used for acetylcholine- and histamine-induced contractions as it is very sensitive to both whereas rat ileum was used for barium chloride-induced contractions. The abdominal cavity was quickly opened and a piece of ileum was isolated. It was placed in a beaker containing Tyrode solution maintained at 37°C. Tissue was cut into pieces of 2-3 cm in length. The distal piece was most preferred and used, being the most sensitive to different spasmogens. The remaining study was carried out on an assembly for isolated tissue i.e. Sherrington revolving drum and an organ bath. The spasms were induced using acetylcholine, histamine and barium chloride.

Submaximal doses of acetylcholine, histamine and barium chloride were selected and different doses of different *shodhit guggul* (like g*ulvel, dashmul, triphala, gomutra shodhit, ashodhit*) were administered. The responses were recorded on the Sherrington recording drum. The same protocol was followed for different processed *gugguls* to evaluate their respective antispasmodic activity. A dose response curve was obtained and then percent inhibition of the contractions produced by submaximal dose of the spasmogen was reported. Results are expressed as mean±standard error mean (SEM), n=4, Student t test

The effect of different processed *guggul* was noted on the length of the intestine travelled by orally administered charcoal in an *in vivo* test. This was expressed as % of small intestine length reached by lower limit of charcoal. The animals were kept fasting for 24 h before the experiment. *Ashodhit guggul* and different processed *guggul* were fed orally 30 min prior to the oral administration of charcoal meal. A 10% w/v solution of animal charcoal in 5% gum acacia was used. 0.5 ml of this suspension was administered orally per mouse irrespective of weight. After 20 min mice were sacrificed by severing the carotids. The stomach and intestine were excised from gastro-esophageal junction to the ileocecal junction. The distance the charcoal meal travelled from the pylorus was measured. As the intestines of the mice used were all of similar length, it was considered justifiable to use the distance travelled by the charcoal meal as an index of intestinal transit. In this way, the intestinal transit was measured for different groups of mice.

Properties of *guggul* have been described as *hridya*, *medoghna* and *mehaghna*^4^. The two important pharmacological properties of g*uggul* are antiinflammatory action^5^ and antihypolipidemic action^6^. Ayurvedic physicians most widely prescribe *guggul* formulations for the treatment of arthritis. *Guggul* is also used for healing bone fractures and inflammations, in cardiovascular disease, obesity and lipid disorders.

Spasms are very common in human beings. Spasms are continuous smooth muscle contractions, may be induced due to endogenous acetyl choline and histamine. They can lead to discomfort, uneasiness and could result into irritation and inflammation of the gastrointestinal tract posing a major health problem to the human being. It could even lead to threatening conditions such as gastritis and inflammatory bowel disorders. Antispasmodics are used to treat such conditions successfully, though they show various side effects such as dry mouth, narrow angle glaucoma, tachycardia, obstructive disease of GI tract.

Acetyl choline-induced spasms are due to muscarinic M_3_ receptor activation, which is a characteristic of vagal stimulation in the body. So mostly all endogenous colic pain like biliary, gastrointestinal, ureter arise due to such acetyl choline-induced spasms. Histamine-induced spasms are mediated by H_1_ receptor activation which is characteristic of allergy producing substances leading to abdominal pain e.g. lead poisoning, uremia, excessive gastric acid or even bile secretion. Barium chloride-induced spasms are not mediated by any receptor but they are mediated by increased Ca^++^ channel entry due to spasmogen or increased phosphodiaesterase activity leading to calcium channel activation.

Percent inhibition of induced spasms by different spasmogens was taken as the parameter to evaluate the antispasmodic activity. Various processed *guggul* namely *gulvel, dashmul and triphala shodhit guggul* showed considerable antispasmodic activity against spasms induced by all three different spasmogens used. This indicates that processed *guggul* possessed antispasmodic activity against spasms of different origin. This explains the possible utility of processed *gugguls* for variety of spasms. *Ashodhit guggul* failed to inhibit the spasms.

 Results indicated that *Ashudha guggul* was not effective in inhibiting the spasms induced by acetylcholine, histamine or barium chloride (Table 1, fig. 1). On the contrary, processed *gugguls* inhibited the spasms induced by these spasmogens. *Gulvel shodhit guggul* showed maximum inhibition of acetylcholine-induced spasms whereas *dashmul shodhit guggul* showed maximum inhibition of histamine-induced spasms. *Triphala shodhit guggul* showed maximum inhibition of barium chloride-induced spasms. *Gulvel shodhit, triphala shodhit* and *dashmul shodhit guggul* have a considerable inhibitory activity against spasms induced by different spasmogens compared to other processed *gugguls*. Entostal, the marketed polyherbal formulation, also exhibited a highly significant inhibitory activity but only in acetylcholine induced spasms. It produced negligible inhibitory activity against barium chloride-induced spasms. When different processed *gugguls* were subjected to charcoal transit along with Entostal, most of the *vishesh shodhit guggul* showed significantly lower charcoal transit indicating that processed *guggul* definitely exhibited better antispasmodic activity than *ashodhit* or even *samanya shodhit guggul* (Table 2, fig. 2). It also showed higher activity than Entostal, which is a marketed antispasmodic preparation.

**TABLE 1:** EFFECT OF *GUGGUL* ON ACETYLCHOLINE, HISTAMINE AND BARIUM CHLORIDE-INDUCED SPASMS IN GUINEA PIG ILEUM

Different *Shodhit guggul* (10 μg/ml)	% Inhibition of Ach- induced spasms in guinea pig ileum	% Inhibition of histamine- induced spasms in rat ileum	% Inhibition of barium chloride- induced response in rat ileum
Control	0	0	0
Entostal	78.33±0.96*	61.25±1.44*	32.15±0.68*
*Ashudha guggul*	8.33±0.93	21.93±0.51	2.66±0.53
*Samanya Shodhit guggul*	25.72±1.77*	43.41±0.93*	22.34±0.61*
*Gomutra Shodhit guggul*	64.82±1.77*	59.91±1.72*	18.09±0.61*
*Dashmul Shodhit guggul*	69.45±1.07*	68.42±1.72*	52.00±0.46*
*Triphala Shodhit guggul*	73.15±1.77*	78.95±1.72*	60.00±0.53*
*Gulvel Shodhit guggul*	76.88±1.76*	87.97±1.71*	63.30±0.53*

Results are expressed as mean±standard error mean (SEM), n=4, student t test, with p value < 0.001 as denoted by*

**Fig. 1:**
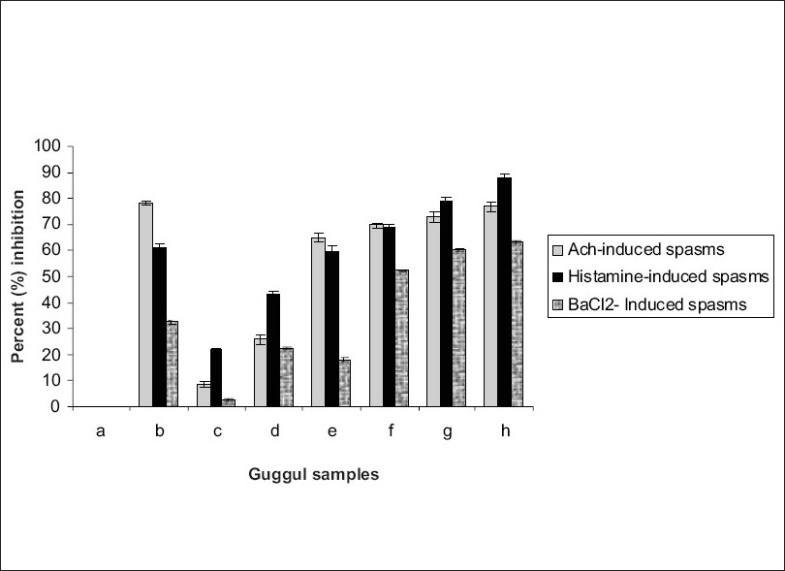
Percent inhibition of Ach, Histamine and BaCl_2_ induced spasms by different guggul samples such as a; Control, b; Entostal, c; *Ashudha guggul*, d; *Samanya shodhit guggul*, e; *Gomutra shodhit guggul*, f; *Dashmul shodhit guggul*, g; *Triphala shodhit guggul*, h; *Gulvel shodhit guggul*

**TABLE 2:** EFFECT OF *GUGGUL* ON CARBON TRANSIT

Different samples of *Shodhit guggul*	Carbon transit mean (SEM) mm (500 mg/ml)	Carbon transit mean (SEM) mm (2000 mg/ml)
Saline	57.62±2.66*	66.87±0.97*
Entostal	20.00±0.41*	24.50±0.29*
*Ashudha Guggul*	50.75±0.48	54.75±0.48
*Samanya Shodhit Guggul*	37.25±0.48*	31.00±0.58*
*Gulvel Shodhit Guggul*	14.00±0.91*	21.25±1.39*
*Gomutra Shodhit Guggul*	15.75±0.52*	20.87±0.65*
*Triphala Shodhit Guggul*	17.25±0.48*	8.50±1.67*
*Dashmul Shodhit Guggul*	15.00±1.47*	24.00±1.67*

Results are expressed as mean±standard error of the mean (SEM), n=4, student t test, with p value < 0.001 as denoted by*

**Fig. 2:**
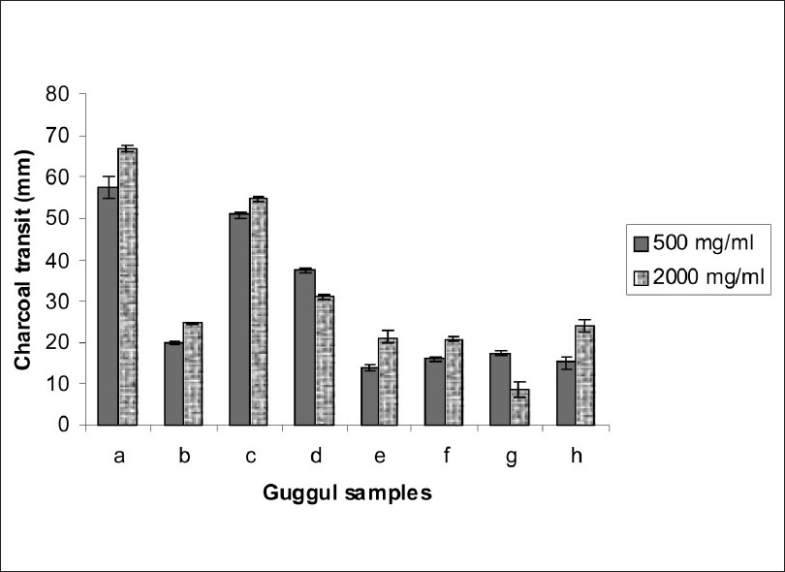
Effect on charcoal transit of different guggul samples such as a; Control, b; Entostal, c; *Ashudha guggul*, d; *Samanya shodhit guggul*, e; *Gomutra shodhit guggul*, f; *Dashmul shodhit guggul*, g; *Triphala shodhit guggul*, h; *Gulvel shodhit guggul*

It is seen that *guggul* inhibited the spasms induced by all Ach, histamine and barium chloride. Thus it may be acting by antagonism of muscarinic M_3_ or histamine H_1_ receptors or it may be simply inhibiting phosphodiaesterase enzyme and consequently inactivating Ca^++^ channels responsible for spasms. The *shodhit guggul* can be thus explored for its activity as antispasmodic agents. Also, *Ashodhit guggul* exhibited very weak antispasmodic activity, indicating that the process of *Shodhan vidhi* is very important to increase the therapeutic activity of a drug. So while manufacturing of ayurvedic medicines, this concept of *Shodhan vidhi* must be considered for safer and better utilization of therapeutic activity of a drug.

When Entostal was evaluated for its antispasmodic activity, it exhibited inhibition of only Ach induced spasms indicating anticholinergic activity against different spasmogens indicating its narrow range antispasmodic activity. As *guggul* processed by different ways exhibited antispasmodic activity both by *in vitro* and *in vivo* methods and against different spasmogens used; further exploration of antispasmodic activity would help *guggul* establish itself as a valuable antispasmodic in therapeutics.
